# Subnational spatial co-distribution of tuberculosis and diabetes mellitus across Ghana’s 16 regions following administrative reorganisation: An ecological analysis

**DOI:** 10.1371/journal.pone.0354405

**Published:** 2026-10-14

**Authors:** Richmond Balinia Adda, Simon Subinleeb Bawa

**Affiliations:** 1 Department of Epidemiology and Biostatistics, School of Public Health, University of Technology and Applied Sciences, Navrongo, Ghana; 2 Navrongo Health Research Centre, Navrongo, Ghana; The University of Georgia, UNITED STATES OF AMERICA

## Abstract

**Background:**

National administrative boundary reorganisations, increasingly common across sub-Saharan Africa, create discontinuities in surveillance data alignment that complicate subnational burden estimation for infectious and non-communicable diseases alike. Ghana’s expansion from 10 to 16 administrative regions in 2019 provides a tractable case for demonstrating how publicly available surveillance and survey data can be used to characterise subnational co-distribution of tuberculosis and diabetes mellitus (DM) under a revised boundary structure. DM is an established risk factor for active TB, yet population-level evidence on their geographic co-distribution at the subnational level in West Africa remains scarce, and no prior study has characterised this co-distribution at the subnational level across Ghana’s current 16-region structure—a gap this study addresses directly by leveraging the post-2019 administrative framework to generate region-specific evidence for programme planning.

**Methods:**

We conducted a cross-sectional ecological analysis using 16 administrative regions in Ghana as the unit of analysis. TB case notification rates (per 100,000 population, 2022) were obtained from the Ghana Health Service’s DHIMS2, with the Ministry of Health 2022 Holistic Assessment and WHO Global Tuberculosis Report 2025 used as independent consistency checks. Regional DM prevalence estimates were drawn from the Ghana STEPS 2017 survey and published scoping literature. Sociodemographic covariates included urbanisation, a DHS-derived wealth index, mean body mass index, and smoking prevalence. Spatial autocorrelation was assessed using Moran’s I with queen contiguity weights. Bivariate associations were estimated using Spearman and Pearson correlations. Negative binomial regression with log-population offset examined the adjusted association between DM prevalence and TB notification rates. A sensitivity analysis excluded the three regions created by the 2019 reorganisation.

**Results:**

TB notification rates ranged from 18.9 to 58.3 per 100,000 (mean 32.0, SD 10.1) and DM prevalence from 1.8% to 6.3% (mean 3.5%, SD 1.3). DM prevalence showed moderate positive spatial autocorrelation (Moran’s I = 0.40, p = 0.004); TB notification rates did not (Moran’s I = −0.16, p = 0.272). Bivariate correlation between the two outcomes was moderately positive (Spearman rho = 0.638, p = 0.008; Pearson r = 0.762, p = 0.0006). In adjusted negative binomial regression, DM prevalence showed a positive but statistically imprecise point estimate (IRR = 3.62; 95% CI: 0.47 to 27.9; p = 0.217), consistent with limited power at n = 16 ecological units.

**Conclusions:**

DM prevalence shows moderate positive spatial autocorrelation across Ghana’s 16 regions, concentrated in the southern urban belt, and is moderately correlated with TB notification rates at the regional level; TB notification rates themselves did not show significant spatial clustering when tested with real-boundary contiguity weights, elevated TB notification alongside low DM prevalence, suggests that drivers other than DM dominate in that zone and that uniform national TB-DM programme approaches may be suboptimal. These methods and findings provide a reproducible template for subnational TB-DM surveillance in other post-reorganisation African programme contexts. Individual-level causal inference is not possible from this ecological design.

## Background

Administrative boundary reorganisations present an underappreciated methodological challenge for subnational disease surveillance. When existing administrative units are subdivided, historical surveillance series are disrupted, regional population denominators require re-estimation, and direct comparison of pre- and post-reorganisation data becomes unreliable without explicit harmonisation. Fragmented surveillance systems resulting from such structural changes are a recognised constraint on subnational epidemiological analysis across sub-Saharan Africa [1]. Ghana’s 2019 constitutional expansion from 10 to 16 administrative regions provides a tractable and well-documented case for examining what subnational epidemiological characterisation is achievable when boundary changes occur and publicly available data sources must be used in combination. Tuberculosis remains a leading infectious cause of death globally, with an estimated 10.7 million new cases and 1.23 million deaths recorded in 2024 [2]. Sub-Saharan Africa carries a disproportionate share of this burden, accounting for approximately one quarter of global TB incidence [2]. In Ghana, approximately 14,000 new and relapse TB cases were notified to the national programme in 2022, corresponding to a national notification rate of approximately 42 per 100,000 population and a case detection rate of 36.7% [3]. The gap between estimated incidence and notified cases suggests substantial under-detection, and geographic variation in diagnostic capacity has been identified as a likely contributing factor [4].

In parallel, the prevalence of type 2 diabetes mellitus has risen across sub-Saharan Africa, driven by urbanisation, dietary change, physical inactivity, and demographic ageing [5,6]. The urban gradient in DM risk is pronounced in West Africa: a multi-country analysis of STEPS survey data from five West African countries, including Ghana, found age- and sex-adjusted T2DM prevalence of 6.2% in urban areas compared with 2.5% in rural areas, representing a relative risk of 2.69 (95% CI: 1.85 to 4.05) [7]. However, country-specific subnational estimates remain limited across the region. Nigeria, the most populous country in West Africa, has reported wide state-level variation in DM prevalence, from 2.3% in rural north-eastern states to 11.2% in urban Lagos, based on pooled national surveys [8], yet comparable state-level TB notification data are not routinely published alongside DM estimates. Côte d’Ivoire and Senegal face similar challenges in aligning TB surveillance data with NCD survey data across subnational administrative units, limiting region-specific TB-DM co-burden assessments [9,10]. This fragmentation in data availability across West African health information systems underscores the value of a standardised analytical framework that can be applied using routine data sources irrespective of boundary structure, and highlights the contribution of the Ghana case to the regional evidence base.

In Ghana, the 2017 STEPS survey estimated national DM prevalence at approximately 3.95% among adults [11], and International Diabetes Federation projections indicate this burden will continue to grow [12]. Subnational heterogeneity in DM prevalence is expected given this urban-rural gradient, but regional estimates aligned to Ghana’s post-2019 boundary structure have not previously been synthesised.

Several lines of evidence suggest that DM increases susceptibility to active TB, with meta-analytic estimates consistently showing an approximately two- to three-fold elevated risk across both global cohort studies and sub-Saharan African studies specifically [13,14]. Proposed biological mechanisms include hyperglycaemia-mediated impairment of macrophage phagocytic function, dysregulation of T-cell subsets, and altered cytokine profiles relevant to containment of *Mycobacterium tuberculosis* [15]. Shared socioeconomic determinants, including poverty, overcrowding, and reduced healthcare access, may also independently elevate risk for both conditions, potentially producing geographic co-clustering in the absence of a direct biological relationship [16].

Published subnational ecological analyses examining the geographic co-distribution of TB and DM at a national scale in sub-Saharan Africa are scarce. Nationwide TB spatial-clustering studies exist for Ethiopia and South Africa [17,18], but comparable analyses incorporating DM as an ecological covariate, or examining the geographic co-distribution of TB and DM jointly, remain rare across the continent and have not been conducted in West Africa. Within Ghana, Amo-Adjei and Tuoyire’s district-level spatial analysis of TB notification rates (2012–2015) predates the 2019 administrative reorganisation from 10 to 16 regions and did not examine DM as a covariate or outcome of interest [19]. The present study extends that work by using the current 16-region administrative structure, by introducing DM prevalence as the primary ecological exposure, and by explicitly addressing the surveillance discontinuity created by the 2019 boundary change as both a methodological challenge and an analytical problem of wider relevance to programmes in other reorganising settings.

Ecological analyses of this kind cannot establish causal relationships at the individual level; the ecological fallacy is a fundamental limitation of aggregate-level inference. Such analyses can, however, serve a descriptive and hypothesis-generating function, identifying where dual burden appears to be concentrated and where spatial clustering is present, to support the design of individual-level studies and inform priority-setting for integrated TB-DM programmes. Critically, they can also demonstrate replicable analytical approaches for characterising subnational disease co-distribution under revised administrative boundary structures, a challenge common to many national health programmes across sub-Saharan Africa, particularly in West African countries such as Nigeria, Côte d’Ivoire, and Senegal where administrative unit changes have occurred or are under consideration. Standardised methods for handling boundary-change uncertainty in subnational surveillance are lacking; the pre-specified sensitivity analysis framework used here, which excludes newly created regions to assess the robustness of observed associations, provides a transparent and transferable approach that other programmes can adopt. This methodological contribution is as important as the substantive findings, because it addresses a practical surveillance problem that will recur as health systems across the region continue to undergo administrative restructuring.

This study therefore aimed to: (i) demonstrate a replicable approach to subnational ecological analysis of disease co-distribution under a recently revised administrative boundary structure, using Ghana’s 16-region system as a case study with direct relevance to other countries undergoing similar administrative changes; (ii) describe the subnational distribution of TB notification rates and DM prevalence across Ghana’s 16 regions; (iii) assess spatial autocorrelation and the degree of geographic overlap between the two outcomes; and (iv) examine the ecological association between DM prevalence and TB notification rates after adjustment for plausible regional sociodemographic covariates.

## Methods

### Study design and setting

We conducted a cross-sectional ecological study using Ghana’s 16 administrative regions as the unit of analysis. Ghana is a lower-middle-income country in West Africa with a population of approximately 32 million as of 2021 [20]. Ghana’s administrative reorganisation from 10 to 16 regions in 2019 created three new regions (Oti, Savannah, and North East) through subdivision of existing regions, disrupting historical surveillance series for those areas. This boundary change is handled explicitly in the analysis through a pre-specified sensitivity analysis excluding the three affected regions, a methodological decision that is described as a transferable analytical approach for similar reorganisation contexts. The explicit handling of boundary-change uncertainty is intended to provide a template that can be adapted by other national programmes facing analogous data alignment challenges, allowing them to generate robust subnational evidence using routinely available surveillance and survey data without requiring primary data collection or substantial additional resources. Analyses are based on the 16-region structure. The ecological design was chosen given the availability of regional-level aggregated surveillance and survey data; individual-level inference is not possible from these data.

### Data sources

*Tuberculosis notification rates.* Regional TB case notification rates (per 100,000 population, 2022) were obtained directly from the Ghana Health Service’s District Health Information Management System (DHIMS2), the national repository of routine TB programme data. The Ghana Ministry of Health 2022 Holistic Assessment of the Health Sector Programme of Work, which separately reports regional case detection rates [3], and the WHO Global Tuberculosis Report 2025 national-level notification counts [2], were used as independent consistency checks on national totals. Because three regions (Oti, Savannah, and North East) were created in 2019 by subdividing former regions, their notification rates carry additional estimation uncertainty, and a sensitivity analysis excluding these three regions is reported separately.

*Diabetes mellitus prevalence.* Regional DM prevalence estimates were drawn from the Ghana STEPS survey 2017, which provides the most recent nationally representative population-level estimates of DM and NCD risk factors in Ghana [11]. Where STEPS did not publish disaggregated subnational estimates for the 16-region structure, supplementary information was drawn from the scoping review by Asante and colleagues (2024), which synthesised 36 studies on DM burden in Ghana and reported the available evidence on regional variation [5]. For Oti, Savannah, and North East, the three regions created in 2019, DM estimates were likewise drawn from these same published sources rather than being statistically modelled from urbanisation, wealth, or other covariates used elsewhere in this analysis; because these source surveys predate the 2019 reorganisation, mapping their findings onto the current boundary structure introduces additional estimation uncertainty, discussed further in the Limitations. The national facility-based prevalence reported in the GHS 2022 Holistic Assessment (0.61% among facility attendees) was noted as an underestimate and used for triangulation only, not as the primary DM measure.

*Sociodemographic covariates.* Regional urbanisation proportions were obtained from the Ghana Statistical Service 2021 Population and Housing Census [20]. The DHS wealth index, mean body mass index among women aged 15–49, and smoking prevalence (men) were extracted from the Ghana Demographic and Health Survey 2022 [21]. These covariates were selected a priori based on their plausible association with both TB and DM, and on data availability at the regional level. Population denominators for rate calculations were from GSS 2022 population projections [20].

### Statistical methods

*Descriptive analysis.* Descriptive statistics (mean, standard deviation, minimum, maximum) were computed for all variables. Normality of TB notification rates and DM prevalence was assessed using the Shapiro-Wilk test (TB: W = 0.925, p = 0.200; DM: W = 0.947, p = 0.443), consistent with approximate normality for both distributions.

*Bivariate correlation.* The bivariate relationship between regional DM prevalence and TB notification rate was assessed using Spearman rank correlation (primary, given the small sample size) and Pearson correlation (secondary). Both statistics are reported with exact two-tailed p-values.

*Spatial autocorrelation.* Spatial autocorrelation in TB notification rates and DM prevalence was assessed using Moran’s I with a queen contiguity spatial weights matrix constructed from real regional boundary polygons (shared borders; geoBoundaries GHA ADM1 administrative boundaries), row-standardised. Given the small number of spatial units (n = 16), Moran’s I estimates and associated p-values should be interpreted with appropriate caution; formal inference used a permutation approach (9,999 permutations, fixed random seed for reproducibility). The full spatial analysis workflow is documented and reproducible using the libpysal and esda packages in Python (version 3.12). Bivariate Moran’s I was additionally computed to characterise the spatial association between the two variables. Moran scatter plots are presented to facilitate visual interpretation of clustering patterns.

*Negative binomial regression.* The association between regional DM prevalence and TB case notification was modelled using negative binomial regression, with TB case counts as the outcome and the natural logarithm of regional population as an offset term. Negative binomial regression was selected a priori over Poisson regression to accommodate potential overdispersion in regional count data, a common feature of small-area count data; the dispersion parameter (alpha = 0.030, SE = 0.028, p = 0.294 versus alpha = 0) was estimated from the fitted model. As detailed in the Results, this estimate was not significantly different from zero, indicating limited evidence of overdispersion beyond Poisson variation in this dataset; the negative binomial specification was nonetheless retained as the pre-specified model, both to preserve the analysis plan and because it nests Poisson as a special case, and results are interpreted with this in mind. All continuous predictors were standardised (mean = 0, SD = 1) to permit comparison of coefficient magnitudes. Incidence rate ratios (IRRs) with 95% confidence intervals are reported.

Given that the analysis is based on only 16 ecological units, the regression model is substantially underpowered for conventional hypothesis testing, and wide confidence intervals are expected. All regression results should be interpreted as exploratory and hypothesis-generating, not as confirmatory evidence of association. Analyses were conducted in Python 3.12 using the statsmodels (version 0.14) and scipy.stats packages. Code and a fully reproducible analysis pipeline, including the regional dataset and spatial boundary files used, are deposited on Figshare (https://doi.org/10.6084/m9.figshare.33415306).

*Sensitivity analyses.* Three pre-specified sensitivity analyses were conducted: (i) exclusion of the three regions created after the 2019 reorganisation (Oti, Savannah, North East), given the greater uncertainty in their boundary-adjusted notification estimates (n = 13); (ii) stratified Spearman correlation among southern and middle-belt regions versus northern belt regions separately; and (iii) comparison of results using Pearson versus Spearman correlation to assess sensitivity to distributional assumptions.

### Ethical considerations

This study involved exclusively publicly available, anonymised, aggregate-level secondary data. No individual participants were recruited, no primary data collection was undertaken, and no human subjects were directly or indirectly involved. The author confirms that all procedures contributing to this work comply with the ethical standards of the Helsinki Declaration of 1975, as revised in 2013.

## Results

### Descriptive characteristics

Data were available for all 16 regions. TB notification rates in 2022 ranged from 18.9 per 100,000 (Oti) to 58.3 per 100,000 (Greater Accra), with a national mean of 32.0 per 100,000 (SD 10.1). Greater Accra and Ashanti, the two most urbanised regions, recorded the highest notification rates (58.3 and 44.1 per 100,000, respectively), consistent with their higher diagnostic capacity. North East and Oti, both newly created and predominantly rural regions, recorded the lowest rates (19.4 and 18.9, respectively). Upper East (41.6) and Northern (35.1) had relatively high rates in the context of the northern belt, which may reflect historically active TB programmes in these regions rather than higher true incidence.

DM prevalence ranged from 1.8% (North East) to 6.3% (Greater Accra), with a national mean of 3.5% (SD 1.3). Regional DM prevalence followed the expected urban gradient, with southern and peri-urban regions showing substantially higher estimated prevalence than northern savannah belt regions. Full regional data are presented in Table 1. The geographic distribution of TB notification rates and DM prevalence across the 16 regions is shown in Fig 1.

**Table 1:** Regional distribution of TB notification rates, DM prevalence, and sociodemographic covariates across Ghana’s 16 administrative regions (2022).

Region	TB Rate (/100k)	DM Prev (%)	Urban (%)	Wealth Index	Mean BMI	Smoking (%)
Greater Accra	58.3	6.3	92.8	4.31	26.1	3.2
Ashanti	44.1	5.2	57.1	3.42	25.4	4.1
Western	38.7	4.8	41.2	2.89	24.8	5.8
Central	31.2	4.6	38.6	2.74	24.6	4.6
Eastern	29.8	3.9	36.4	2.68	24.2	5.2
Volta	26.4	3.4	31.3	2.31	23.5	4.8
Oti	18.9	2.1	18.7	1.87	22.4	6.1
Bono	33.5	3.8	42.1	2.95	24.1	5.5
Bono East	24.1	3.1	28.9	2.42	23.3	6.3
Ahafo	29.2	3.5	34.8	2.78	23.7	5.9
Western North	22.7	2.8	22.1	2.13	22.8	7.1
Northern	35.1	2.4	29.4	1.94	22.1	8.2
Savannah	28.3	2.0	19.8	1.62	21.6	8.9
North East	19.4	1.8	17.3	1.58	21.4	8.4
Upper East	41.6	2.6	27.9	2.03	22.3	7.3
Upper West	30.8	2.9	25.6	2.11	22.5	7.8
**National mean**	32.0	3.5	35.3	2.49	23.4	6.2
**National SD**	10.1	1.3	18.5	0.71	1.4	1.6

*TB rate: new and relapse TB cases notified per 100,000 population (2022); source: GHS DHIMS2, with the MoH 2022 Holistic Assessment and WHO Global Tuberculosis Report 2025 used as independent consistency checks. DM Prev: estimated regional DM prevalence (%) from Ghana STEPS 2017 and Asante et al. 2024. Urban: proportion of population residing in urban areas (GSS 2021 PHC). Wealth Index: DHS composite wealth index (mean, 1–5 scale). BMI: mean body mass index (kg/m²) among women 15–49 (DHS 2022). Smoking: prevalence of current smoking among men (%), DHS 2022/ STEPS 2017. Rows are ordered by geographic zone. National mean and SD computed across 16 regions.*

**Fig 1:**
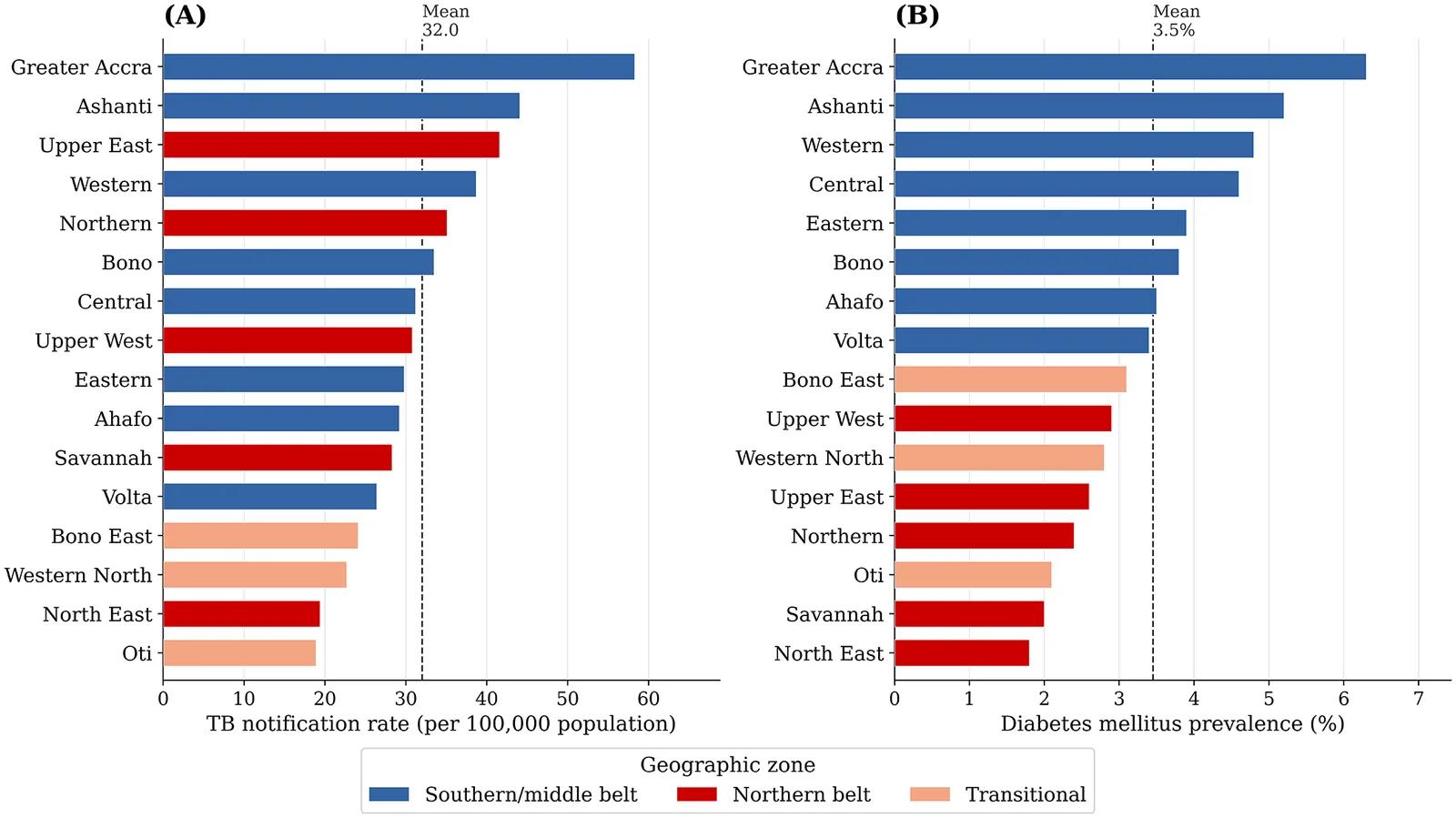
Subnational distribution of TB notification rates (A) and DM prevalence (B) across Ghana’s 16 administrative regions (2022). Bars are sorted by ascending value within each panel. Dashed vertical lines indicate the national mean. Regions are colour-coded by geographic zone: blue = southern/middle belt; red = northern savannah belt; orange = transitional. TB notification rates are per 100,000 population. DM prevalence is the estimated percentage of adults with DM. Sources: GHS DHIMS2 (TB), with the MoH 2022 Holistic Assessment used for consistency checking; Ghana STEPS 2017 and Asante et al. 2024 (DM); Ghana DHS 2022 and GSS 2021 PHC (covariates).

### Spatial autocorrelation

DM prevalence showed moderate positive spatial autocorrelation across the 16 regions (Table 2): Moran’s I was 0.40 (z = 3.14, p = 0.004), indicating that neighbouring regions tended to have more similar DM prevalence than would be expected by chance. TB notification rates, in contrast, showed no significant spatial autocorrelation when tested using a queen contiguity matrix built from real regional boundaries (Moran’s I = −0.16, z = −0.65, p = 0.272); TB notification does not exhibit the same geographic clustering pattern as DM prevalence at the regional level. Bivariate Moran’s I, reflecting the spatial co-clustering of TB and DM, was 0.06 (z = 0.45, p = 0.322) and was not statistically significant. These patterns are visualised in Fig 2, which shows the Moran scatter plot of DM prevalence against its spatial lag. Southern and peri-urban regions (Greater Accra, Ashanti, Western) cluster in the high-high quadrant for DM, while several northern and transitional regions cluster in the low-low quadrant.

**Table 2:** Moran’s I statistics for spatial autocorrelation of TB notification rates, DM prevalence, and their bivariate spatial association across Ghana’s 16 regions (2022).

Variable	Moran’s I	Z-score	p-value (perm.)
TB notification rate	−0.16	−0.66	0.272
DM prevalence	0.40	3.14	0.004
Bivariate TB × DM	0.06	0.45	0.322

*Spatial weights: queen contiguity matrix derived from real regional boundary polygons (shared borders; geoBoundaries GHA ADM1), row-standardised. Inference based on 9,999 conditional permutations (fixed random seed, reset independently per statistic for reproducibility). Given n = 16 spatial units, estimates should be interpreted with appropriate caution.*

**Fig 2:**
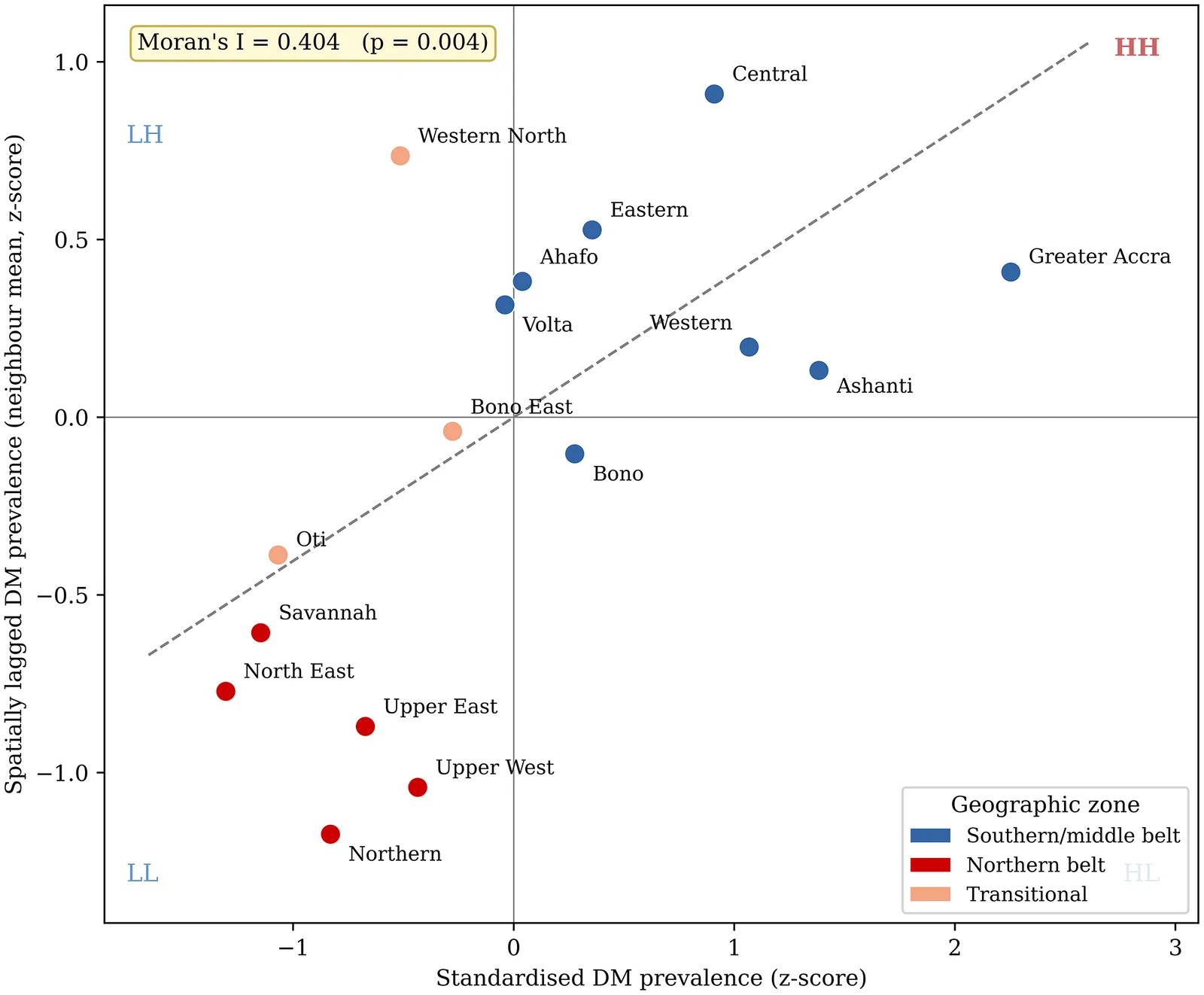
Moran scatter plot of regional DM prevalence and its spatial lag across Ghana’s 16 regions. Each point represents one region. The x-axis shows standardised DM prevalence; the y-axis shows the spatially lagged (neighbourhood mean) standardised DM prevalence computed from a queen contiguity weights matrix derived from real regional boundary polygons (shared borders; geoBoundaries GHA ADM1). The dashed line is the Moran regression slope (slope = Moran’s I). Quadrant labels: HH = high-high cluster; LL = low-low cluster; HL/LH = spatial outliers. Reference lines at zero for both axes. Colour coding as in Fig 1. Moran’s I for DM was 0.40 (z = 3.14, permutation p = 0.004, 9,999 permutations).

With only 16 spatial units, the statistical properties of Moran’s I are less reliable than in analyses with larger numbers of areal units, and these results should be interpreted with commensurate caution. This caution applies with particular force to the non-significant TB and bivariate estimates, which indicate that this analysis did not detect spatial clustering at conventional significance thresholds rather than establishing its absence. Table 2 reports the Moran’s I statistics for TB, DM, and their bivariate spatial association.

### Bivariate association between DM and TB

The Spearman rank correlation between regional DM prevalence and TB notification rate was 0.638 (p = 0.008, two-tailed), and the Pearson correlation was 0.762 (p = 0.0006). These results indicate a moderately strong positive ecological association between the two variables at the regional level. The scatter plot (Fig 3) shows that Greater Accra and Ashanti, the two most urbanised regions, contribute substantially to this correlation through high values on both axes, and a degree of leverage from these observations should be noted. Excluding Greater Accra, the Spearman correlation was 0.56 (p = 0.030), indicating the association was not entirely attributable to a single influential observation.

**Fig 3:**
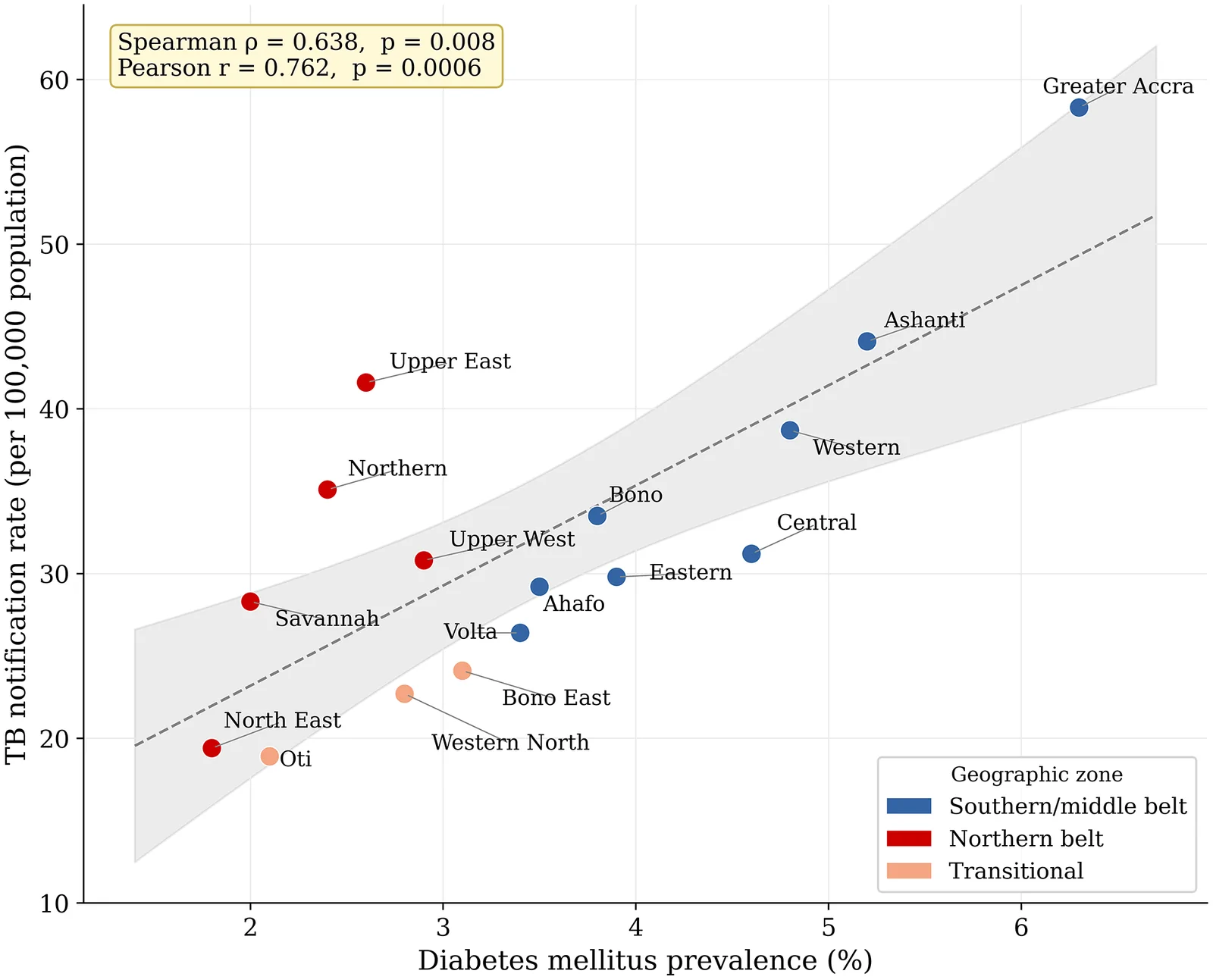
Regional DM prevalence and TB notification rate across Ghana’s 16 regions (2022), with fitted linear regression line. Each point represents one region, labelled by name and coloured by geographic zone. The dashed line is the OLS regression line fitted for visualisation purposes; the shaded band is the 95% confidence envelope. Spearman rank correlation: rho = 0.638, p = 0.008. Pearson correlation: r = 0.762, p = 0.0006. These are ecological-level associations; individual-level inference cannot be drawn from this figure.

### Negative binomial regression

Results of the adjusted negative binomial regression are presented in Table 3. The overall model was statistically significant by likelihood ratio test (LLR p = 0.003). The estimated dispersion parameter alpha was 0.030 (SE 0.028), which was not significantly different from zero (p = 0.294), indicating limited evidence of overdispersion beyond Poisson variation in this dataset; the negative binomial specification was nonetheless retained as the pre-specified model, and results should be interpreted with this in mind. The AIC was 196.5.

**Table 3:** Adjusted negative binomial regression of TB case notification rates on DM prevalence and sociodemographic covariates across Ghana’s 16 regions (2022).

Predictor (standardised)	Coef.	SE	IRR (95% CI)	p-value
DM prevalence	1.29	1.04	3.62 (0.47, 27.9)	0.217
Urban population %	−0.21	0.54	0.81 (0.28, 2.35)	0.702
Wealth index (DHS)	0.44	0.68	1.55 (0.41, 5.84)	0.520
Mean BMI	−1.34	1.37	0.26 (0.02, 3.86)	0.328
Smoking prevalence	−0.03	0.26	0.97 (0.59, 1.61)	0.907
**Dispersion (alpha)**	0.030	0.028	–	0.294
**LLR p-value (model)**	–	–	–	0.003

*All continuous predictors standardised (mean = 0, SD = 1). Outcome: regional TB case count. Offset: natural log of regional population. IRR: incidence rate ratio = exp(coefficient). 95% CI: Wald confidence interval. LLR: log-likelihood ratio test against the null model. Dispersion parameter alpha estimated by maximum likelihood. Given n = 16, these estimates are exploratory and carry very wide uncertainty; statistical non-significance for individual predictors should not be interpreted as absence of effect.*

In the adjusted model, the point estimate for DM prevalence was positive and in the expected direction (IRR = 3.62; 95% CI: 0.47 to 27.9; p = 0.217), but with a very wide confidence interval that included values below and substantially above 1.0, reflecting the limited power of this analysis. None of the individual predictors reached conventional statistical significance in the adjusted model, including urbanisation (IRR = 0.81; 95% CI: 0.28 to 2.35; p = 0.702), wealth index (IRR = 1.55; 95% CI: 0.41 to 5.84; p = 0.520), mean BMI (IRR = 0.26; 95% CI: 0.02 to 3.86; p = 0.328), or smoking prevalence (IRR = 0.97; 95% CI: 0.59 to 1.61; p = 0.907). The absence of individual statistical significance in these predictors is expected given the very small sample size and should not be interpreted as evidence of no association.

### Sensitivity analyses

As a pre-specified sensitivity analysis addressing the surveillance discontinuity introduced by the 2019 reorganisation, excluding the three newly created regions (Oti, Savannah, North East; n = 13) attenuated the Spearman correlation to 0.434 (p = 0.138), suggesting attenuated association and greater uncertainty in the smaller subset. This attenuation is consistent with reduced variability in both variables when the most rural and lowest-burden regions are removed, rather than necessarily indicating a spurious association in the full dataset.

Stratified correlations by geographic zone showed a stronger positive association among southern and middle-belt regions (n = 11; Spearman rho = 0.973, p < 0.001) than among the northern belt alone (n = 5; Spearman rho = 0.700, p = 0.188), with the latter analysis limited by very small sample size. This pattern suggests that the overall bivariate correlation is driven primarily by the marked contrast between southern urban regions with high DM and TB and northern rural regions with lower DM but heterogeneous TB notification rates.

## Discussion

### Principal findings

This subnational ecological analysis described the geographic distribution of TB notification rates and DM prevalence across Ghana’s 16 administrative regions. DM prevalence showed moderate positive spatial autocorrelation, whereas TB notification rates did not; the two outcomes nonetheless showed a moderately strong positive bivariate ecological association at the regional level (Spearman rho = 0.638, p = 0.008). In an adjusted negative binomial regression model, DM prevalence showed a positive but statistically imprecise point estimate (IRR = 3.62; 95% CI: 0.47 to 27.9), consistent with the expected direction of association but not informative about the magnitude of effect at this ecological scale. Southern urbanised regions, particularly Greater Accra and Ashanti, showed high values on both TB notification and DM prevalence, while northern and transitional regions generally showed lower DM prevalence alongside variable TB notification rates. The divergent pattern in the northern belt, where Upper East and Northern regions show relatively elevated TB notification rates alongside low DM prevalence, is a substantive finding that challenges a uniform TB-DM narrative and has implications for geographically differentiated programme design.

### Comparison with existing literature

The bivariate ecological pattern observed in this study is broadly consistent with the established literature on TB-DM comorbidity. Jeon and Murray’s meta-analysis of 13 observational studies found a relative risk of approximately 3.1 for active TB among individuals with DM in cohort studies [13], and a systematic review restricted to sub-Saharan African studies estimated a pooled odds ratio of 2.77 (95% CI: 1.90 to 4.05) [14], confirming the directionality of this association in the regional context. More recent meta-analyses in the African context have reported DM prevalences among TB patients ranging from approximately 4% to 19%, depending on setting and diagnostic criteria [22]. These individual-level estimates provide biological plausibility for the ecological co-distribution observed here, though ecological correlations cannot be equated with individual-level risk.

At the subnational level in sub-Saharan Africa, ecological analyses of TB-DM geographic co-distribution remain rare. Studies in South Africa and Ethiopia have documented urban clustering of both TB and DM burden, broadly consistent with the DM spatial pattern observed here [17,18]. The Ghana data contribute a West African example to this sparse comparative literature and illustrate that the urban-southern concentration of TB-DM co-burden is not specific to southern or eastern African settings.

At the country level, Amo-Adjei and Tuoyire’s district-level spatial analysis of Ghana TB data from 2012 to 2015 documented significant geographic clustering of TB notification rates, with higher rates in coastal and urban districts [19]. This earlier district-level finding for TB is not directly replicated by the present regional-level analysis, which did not find significant spatial autocorrelation in TB notification rates once tested with a real-boundary contiguity matrix; the difference may reflect the coarser regional (rather than district) scale used here, among other possibilities. The positive spatial autocorrelation in DM prevalence has not, to our knowledge, been previously characterised in Ghana at the regional level, though an urban-rural gradient in DM prevalence is well established in the literature on West African NCD epidemiology [5,7].

In the wider West African context, Nigeria’s National Tuberculosis and Leprosy Control Programme has reported subnational heterogeneity in case notification, with Lagos, Kano, and Oyo states accounting for a disproportionate share of notified cases [8]. However, a recent scoping review of TB-DM comorbidity research in Nigeria identified only one study examining geographic overlap at the subnational level, highlighting the general scarcity of such analyses across the region [23]. Côte d’Ivoire and Senegal have similar gaps: despite national-level estimates of TB incidence and DM prevalence, published subnational spatial analyses of their co-distribution are absent [9,10]. This regional evidence gap reinforces the importance of the Ghana case as a contribution to the broader West African literature and underscores the need for comparable analyses in neighbouring countries.

Comparable TB-DM research also exists outside sub-Saharan Africa, in North Africa and the wider Eastern Mediterranean region, though it has followed a different design tradition. A nationwide facility-based screening study in Egypt found a diabetes case-detection rate of 2.09% among tuberculosis patients screened without a prior DM history, with the highest screening yield among patients aged 40 years and above [24]. Findings of this kind are broadly consistent with the elevated TB-DM comorbidity documented across the region, but, like most existing African TB-DM literature, are individual-level and facility-based rather than subnational and ecological; we are not aware of any published subnational spatial analysis of TB-DM co-distribution from a North African country. The present study’s ecological, region-level approach therefore complements rather than duplicates this individual-level North African evidence, and the methodological gap identified for West Africa above extends to North Africa as well.

### Mechanisms and contextual interpretation

The ecological correlation between regional DM prevalence and TB notification rate could reflect at least three distinct mechanisms. First, if DM genuinely increases individual susceptibility to active TB through immune dysregulation [15], then regions with higher DM burden would be expected to have higher TB incidence and, consequently, higher notification (assuming detection rates are not systematically different). Second, both conditions share socioeconomic determinants: urbanisation, poverty, and limited healthcare access independently increase risk for both TB and DM, and their co-clustering may partly reflect these shared upstream factors rather than a direct DM-to-TB biological pathway [16]. Third, TB notification rates are influenced by diagnostic capacity and health-seeking behaviour, both of which are higher in urban regions, which also tend to have higher DM prevalence. This creates a potential confounding pathway: urbanisation could independently elevate both notification rates (through better detection) and DM prevalence (through lifestyle factors), producing an ecological association without any individual-level causal link.

The divergent pattern in the northern belt is notable and merits closer examination. Upper East and Northern regions had relatively high TB notification rates (41.6 and 35.1 per 100,000, respectively) relative to their DM prevalence (2.6% and 2.4%), suggesting that in these regions, factors other than DM are more important ecological drivers of TB notification. Several such factors are documented for these settings specifically. TB/HIV co-infection is a recognised driver of TB burden in northern Ghana: a facility-based study in the Northern Region reported an ongoing burden of TB/HIV co-infected patients and noted that the region also has the highest multidimensional poverty rate of Ghana’s regions (80.8% of the population, poverty index 0.491), a socioeconomic profile plausibly linked to both TB exposure and delayed case detection [25]. Historically intensive TB case-finding activity in the Upper East and Northern regions, sustained through longstanding donor- and NGO-supported control programmes in the area, may also elevate measured notification independently of true incidence, consistent with the possibility raised earlier that some of the northern belt’s apparent TB burden reflects detection effort rather than transmission intensity. Cross-border population movement with Burkina Faso, which shares a land border with all three northern-belt regions, is a further plausible contributor that has not been quantified in the available surveillance data. This heterogeneity reinforces the importance of disaggregated subnational analysis and cautions against assuming that the TB-DM association observed at the aggregate national level applies uniformly across geographic zones.

### Implications

These findings provide a spatial evidence base for characterising where TB and DM co-burden appears to be most concentrated in Ghana, which is a necessary preliminary step before targeting integrated screening or case management interventions. The WHO collaborative framework for TB and DM recommends bidirectional screening in high-burden settings [26], and systematic reviews of implementation experience in Africa suggest that such programmes are feasible in primary care settings but require dedicated coordination between TB and NCD services, which are typically organised under separate programmatic structures in Ghana [22]. Based on these findings, several specific policy actions for the Ghana Health Service and other national TB programmes in the region warrant consideration. First, a phased geographic approach to integrated TB-DM screening is recommended, with initial implementation in Greater Accra and Ashanti regions, where the dual burden is highest, before expanding to other regions with intermediate burden such as Western and Central. This would allow the health system to test feasibility, refine protocols, and build capacity in a targeted manner before national scale-up. Second, the Ghana Health Service should consider establishing linked TB-DM patient registries at the regional level, starting with the four southern regions where case volumes are highest, to enable routine monitoring of comorbidity prevalence and treatment outcomes. Such registries would provide the individual-level data needed to confirm the ecological associations observed here and to track trends over time. Third, the divergent pattern observed in the northern belt, where Upper East and Northern regions have elevated TB notification alongside low DM prevalence, suggests that TB programme investments in these regions should continue to prioritise traditional TB risk factors such as HIV co-infection, poverty alleviation, and improved case-finding, rather than diverting limited resources to DM screening that may yield low yield. This geographically differentiated approach would allow more efficient use of scarce programme resources. Fourth, at the national level, the Ministry of Health should commission a dedicated subnational DM prevalence survey aligned with the 16-region structure to improve the precision of the regional estimates used in this analysis and to provide a baseline for future trend monitoring.

For other national TB programmes in West Africa and across the continent, these findings illustrate that routine surveillance data, when combined with NCD survey data and spatial analytical methods, can generate actionable evidence for programme planning even in settings with recent administrative changes. The geographical stratification approach proposed here, prioritising integrated screening in high-dual-burden areas while maintaining traditional TB interventions in areas where DM is not a dominant driver, offers a framework that can be adapted to other country contexts based on their own subnational data patterns.

Beyond Ghana, these findings illustrate an approach that national TB programmes in other post-reorganisation settings can replicate using routinely available data. The combination of DHIMS2-equivalent surveillance outputs, national NCD survey data, and freely available administrative boundary files (GADM) produces a spatial evidence base for TB-DM co-distribution that does not require primary data collection or additional resources. Countries that have recently undergone administrative boundary changes, including Nigeria, Ethiopia, and several francophone West African states, face analogous data alignment challenges, and the pre-specified sensitivity analysis framework used here provides a replicable template for managing that uncertainty transparently. The WHO collaborative framework for TB and DM [26] calls for integrated surveillance and screening, but does not provide guidance on how to implement this when subnational boundary structures are in flux; this analysis illustrates one practical response to that gap.

### Limitations

Several limitations should be noted. First, and most fundamentally, this is an ecological study; associations observed at the regional level do not necessarily reflect individual-level associations, and the ecological fallacy remains a central and irreducible limitation. Second, with n = 16 regions, the analysis is severely underpowered for regression modelling, and all point estimates carry very wide confidence intervals. The negative binomial regression should be considered exploratory. Third, TB notification rates reflect detected, not true, TB incidence, and detection rates vary substantially across regions (national CDR 36.7% in 2022 [3]), introducing non-random measurement error that could bias the apparent ecological associations. Regions with better healthcare infrastructure may have higher notification rates not because they have more TB, but because they find more cases; the same regions also tend to have higher DM prevalence, which could inflate the observed correlation. Fourth, DM prevalence estimates at the regional level are drawn from STEPS (2017) and the scoping literature, neither of which provides fully comparable, contemporaneous estimates for all 16 regions under the post-2019 boundary structure. Fifth, DM prevalence estimates for the three regions created by the 2019 reorganisation (Oti, Savannah, North East) were drawn from published empirical sources, primarily the scoping review by Asante and colleagues (2024) supplemented by the Ghana STEPS 2017 survey, rather than being statistically imputed or modelled from urbanisation, wealth, or other covariates used elsewhere in this analysis. This distinction matters for interpreting the observed associations: because these DM values were not constructed from the sociodemographic covariates included in the regression model, their correlation with urbanisation and with TB notification rates is not a circular artefact of the estimation method itself. Nonetheless, because the underlying source surveys predate the 2019 administrative reorganisation, mapping their region-specific findings onto the current 16-region structure carries some residual estimation uncertainty, which is why these three regions are treated separately in the pre-specified sensitivity analysis. This boundary-change limitation is common to any country that has undergone administrative reorganisation and is not specific to Ghana; the sensitivity analysis approach used here, excluding affected regions to assess robustness, is proposed as a general, replicable strategy for managing this uncertainty in comparable settings. We encourage researchers and programme managers in other post-reorganisation contexts to adopt this approach as a standard practice when conducting subnational analyses using routine data, as it provides a transparent assessment of how boundary changes affect observed associations and helps avoid over-interpretation of estimates derived from newly created units. Sixth, important potential confounders at the regional level, including HIV prevalence (GHS 2022 report provides HIV data [3]), healthcare facility density, and population age structure, could not be fully accounted for. Finally, although the spatial weights matrix used here was constructed from real regional boundary polygons rather than an approximation, Moran’s I at n = 16 remains sensitive to the precise specification of the neighbour structure; alternative weights definitions (e.g., distance-based or k-nearest-neighbour) were not systematically compared and could plausibly yield different estimates, particularly for TB notification rates, where the spatial autocorrelation estimate did not reach statistical significance.

### Future research

The primary evidence gap at the global level is the absence of comparable subnational ecological analyses from other West African high-burden countries against which Ghana’s patterns could be benchmarked. Within Ghana, the most important next methodological step is the development of linked individual-level data connecting TB diagnosis with DM status at the population level. Future studies using routinely collected clinical data from TB treatment centres, with systematic DM screening at point of TB diagnosis, would provide individual-level evidence on the DM-TB association in this setting and build on the spatial baseline described here. Prospective cohort studies following DM patients in NCD clinics for incident TB would address questions of temporal ordering and risk magnitude. District-level spatial analyses using DHIMS2 data, once harmonised for post-2019 boundaries, would provide finer-grained targeting information than the regional-level analysis presented here. At the surveillance system level, the more immediate operational gap is the absence of a regional registry of comorbid TB-DM cases that would allow the ecological patterns described here to be tracked prospectively across the reorganised boundary structure. Comparative analyses across other high-burden West African countries would help contextualise Ghana’s subnational patterns within the wider regional burden and strengthen the cross-national methodological contribution of this approach.

## Conclusions

This ecological analysis provides the first subnational characterisation of TB notification rates and DM prevalence at the regional level across Ghana’s 16 administrative regions under the post-2019 boundary structure—a level of geographic detail that is directly actionable for regional health directors and programme managers, and proposes a replicable analytical framework for countries where administrative reorganisation has disrupted surveillance continuity. DM prevalence showed moderate positive spatial autocorrelation, though TB notification rates did not, and a moderately strong positive bivariate ecological association was observed between the two outcomes (Spearman rho = 0.638). The divergent pattern in the northern belt, elevated TB notification with relatively low DM prevalence, suggests that socioeconomic and infectious disease co-determinants dominate over DM as an ecological driver in that zone, and implies that geographically differentiated rather than uniform TB-DM programme approaches are warranted. These findings are descriptive and cannot support individual-level causal inference, but they provide a spatial evidence base for programme prioritisation and a methodological template for comparable subnational surveillance analyses across sub-Saharan Africa. The analytical framework demonstrated here, combining routine TB notification data, NCD survey estimates, spatial autocorrelation methods, and explicit sensitivity analysis for boundary-change uncertainty, can be replicated by national TB programmes in other countries that have undergone administrative reorganisations, including Nigeria, Côte d’Ivoire, and Senegal, to generate local evidence for geographically differentiated TB-DM programme planning using existing data resources.
